# Prevalence and Risk Factors of Reduced Bone Mineral Density in Systemic Lupus Erythematosus Patients: A Meta-Analysis

**DOI:** 10.1155/2019/3731648

**Published:** 2019-02-20

**Authors:** Jumei Xia, Ran Luo, Shuiming Guo, Yi Yang, Shuwang Ge, Gang Xu, Rui Zeng

**Affiliations:** ^1^Department of Nephrology, Tongji Hospital Affiliated with Tongji Medical College, Huazhong University of Science and Technology, 1095 Jie Fang Avenue, Wuhan 430030, China; ^2^Department of Nephrology, PLA 474 Hospital, Urumqi 830000, China

## Abstract

**Background:**

We aimed to conduct a meta-analysis concerning the frequency and risk factors of reduced bone mineral density (BMD) in systemic lupus erythematosus (SLE) with evidence from published studies.

**Methods:**

A comprehensive literature search was conducted based on the EMBASE, Web of Science, PubMed, and Cochrane Library databases up to March 5th, 2017. Eligible studies reported any prevalence of reduced BMD in SLE patients. All risk factors with odds ratios or risk ratios associated with reduced BMD were extracted.

**Results:**

71 reports with 33527 SLE patients were included. Low BMD, osteopenia, and osteoporosis at any site were presented, respectively, in 45%, 38%, and 13% of the SLE patients. The prevalence of osteoporosis increased with the advancing of age, while U-shaped associations between age and the prevalence of low BMD and osteopenia were found. Lumbar spine was indicated to have higher prevalence of osteoporosis. Age, disease duration, drugs use, and many other factors were identified as predictors of reduced BMD.

**Conclusion:**

Low BMD, osteoporosis, and osteopenia appeared to be prevalent in patients with SLE. Risk factors of reduced BMD were various.

## 1. Introduction

Nowadays the long-term complications of systemic lupus erythematosus (SLE) have become great concerns as the survival of SLE has improved dramatically [1]. Publications have shown that patients with SLE have an increased risk of developing reduced bone mineral density (BMD) [2, 3]. The frequency of osteopenia according to WHO criteria is reported to be from 24% to 74% [4, 5] and osteoporosis is from 1.4% to 68.7% [3, 6, 7] in SLE patients. However, most of them are single-center studies and the outcomes vary widely.

Risk factors associated with decreased BMD are still under debate in SLE patients. SLE occurs commonly in females [8, 9]. Women are known to have high prevalence of osteoporosis and osteoporotic fractures [10]. Long-term use of corticosteroid, immunosuppressives, and other drugs, SLE disease damage, and menopause status all might have an effect on bone loss. Osteoporosis is a prevalent complication of SLE and it may lead to increased morbidity and mortality [11, 12]. The reported risk factors from different studies differ greatly [13–15].

The aim of this study was to conduct a meta-analysis concerning the frequency and risk factors of osteopenia, osteoporosis, or low BMD in SLE patients with evidence from published studies.

## 2. Materials and Methods

This study was conducted according to the Preferred Reporting Items for Systematic Reviews and Meta-Analysis (PRISMA) Checklist [16] (Supplementary 1. S1 file).

### 2.1. Literature Search Strategy

We performed a comprehensive literature search based on the PubMed, Web of Science, Cochrane Library, and EMBASE up to March 5th, 2017. We used the keywords including “(Systemic Lupus Erythematosus or SLE)” and “(risk factors or outcomes or Prevalence)” and “(bone mineral density or bone density or Osteoporosis or Osteopenia or Fracture)” (Supplementary 2. S2 file). Moreover, we hand-searched the reference lists of all identified eligible papers and relevant narrative reviews for additional relevant studies. Titles and abstracts were screened to identify potentially relevant studies; of these, full texts were reviewed. The full text of an article was assessed if there was any doubt to the eligibility of it. Two of the authors (Y.Y. and J.M.X) independently undertook literature search and study selection using a standardized approach. Reviewers were not blinded to study authors or outcomes. Any inconsistencies were resolved by discussion or by consulting a third author (S.W.G.).

### 2.2. Inclusion and Exclusion Criteria

We included published original articles and data on prevalence of decreased bone density, osteoporosis, or osteopenia in SLE patients. There was no restriction of language. Letter, review, conference abstract, editorial material, comment, case report, meta-analysis, and book chapter were excluded. If two articles from the same population reported different data, we included both of them. Otherwise, if the articles reported the same prevalence, we included the article with the larger sample size. In a longitudinal study that reported prevalence of decreased bone density in different time points, we included the baseline data.

### 2.3. Data Extraction

We extracted the information of interest by two authors (Y.Y. and J.M.X) from each study including study characteristics (study group name, publication year, sample size, age at baseline, female%, SLEDAI, SLICCR/ACR/SDI, BMI, proportion of postmenopausal status, proportion of corticosteroid ever user, mean cumulative corticosteroid dose, age at disease onset, SLE duration, and SLE diagnostic criteria) and any prevalence (data to calculate it) of low BMD (defined as the prevalence of low BMD, or the sum of osteoporosis and osteopenia, or 100%-normal; or if only osteoporosis or osteopenia was reported, defined as the prevalence of osteoporosis or osteopenia) in SLE patients at any part of body. We classified the prevalence according to different sites and different degree of decreased bone density. If a study measured three or more sites of any one patient and gave whole prevalence of osteoporosis, osteopenia, or low BMD for this population or reported the prevalence without stating the sites, we classified this prevalence as “at any site.”

### 2.4. Statistical Analysis

Heterogeneity of prevalence across included studies was examined using *χ*^2^ based on Q-statistical test and quantified by I^2^ index. Roughly, heterogeneity was considered significant at P<0.10 and Higgins I^2^ values of 25%, 50%, and 75% were considered low, moderate, and high inconsistencies. Results of studies were pooled by random-effects models in the presence of high heterogeneity among our studies. All analyses and graphs were made using Stata 10.0 (College Station, TX, USA) and GraphPad Prism 6. P values < 0.05 by two-tailed test were considered significant.

## 3. Results

### 3.1. Study Search and Basic Characteristics

**Figure 1:**
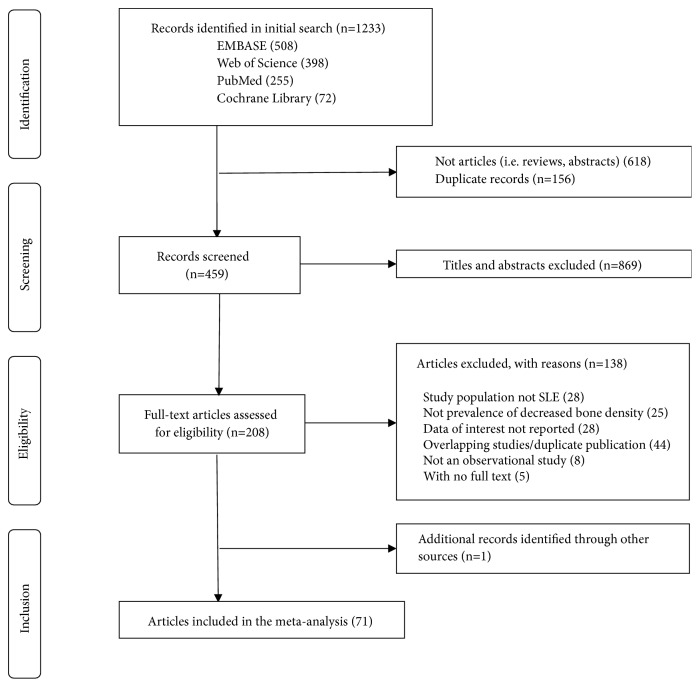
Flow chart of the systematic review.

We identified 1233 articles by systematic electronic searches; 70 of those were eligible for this meta-analysis. And 1 study was included through hand-searching the reference lists of all identified eligible papers (flow diagram for selection of studies as Figure 1). References and characteristics of studies included in this meta-analysis were listed in Supplementary 3. S3 file and Supplementary 4. S4 file, respectively. 33527 SLE patients (female: 90.2%, postmenopausal: 31.6%) with an overall average age of 43.5 years were eligible for inclusion. The mean disease duration was 8.5 years, the mean SLEDAI score was 4.7, and mean SLICC damage index was 1.1. Percentage of steroids ever used was demonstrated in 36 studies and the mean percentage was 78.8%. Mean cumulative dose of steroids was 20.6 gram. More than half the studies (38/71) were published after 2010. Of the included studies, prevalence of low BMD, osteopenia, and osteoporosis was, respectively, reported in 57, 60, and 46 studies; decreased prevalence of bone density of lumbar spine, femur, total hip, and any one site was respectively reported in 32, 20, 22, and 52 studies.

### 3.2. Prevalence of Decreased Bone Density in Different Sites and Patients

The pooled prevalence of low BMD, osteopenia, and osteoporosis at different skeletal sites for all patients and postmenopausal and premenopausal patients was represented in Table 1. The heterogeneity was high for most of the subgroup analyses. The prevalence of low BMD, osteopenia, and osteoporosis was 45% (38, 51), 38% (31, 45), and 13% (11, 16), respectively. Compared to premenopausal patients, postmenopausal patients had relatively higher prevalence of low BMD in all sites. Lumbar spine was indicated to have higher prevalence of osteoporosis.

### 3.3. Exploring the Potential Risk Factors

**Figure 2:**
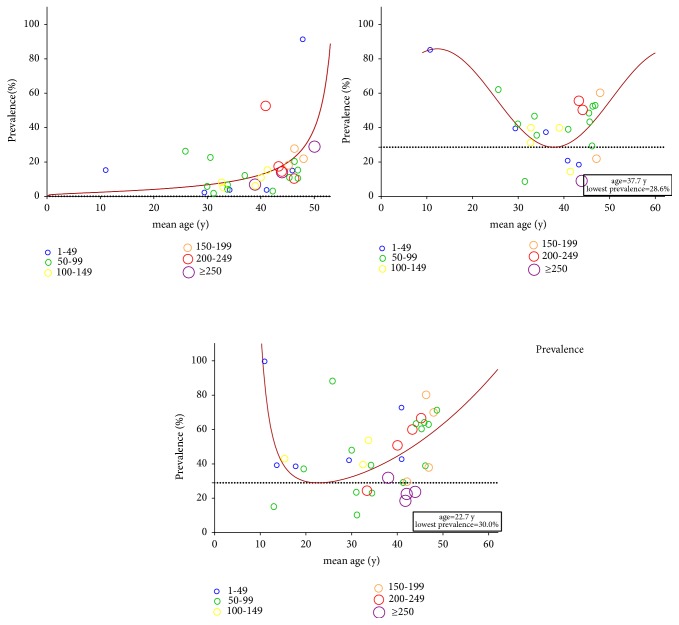
Prevalence of osteoporosis, osteopenia, and low BMD at any site by age for all SLE patients from the literature search.

Figure 2 showed the three models of prevalence by age for osteoporosis, osteopenia, and low BMD at any site. The prevalence of osteoporosis increased with the advancing of age, while U-shaped associations between age and the prevalence of low BMD and osteopenia were found. Age might have influence on bone health.

The association between potential risk factors and low BMD, osteoporosis, and fracture in SLE patients from the literature search was shown in Table 2. Postmenopausal status, non-Afro-Caribbean, higher BMI z score, number of deliveries, ever taken prednisolone >10 mg/day, and maximal dosage of >50 mg/day of oral corticosteroids were significantly associated with low BMD, while menopause, disease duration, and prednisone use were associated with osteoporosis. The risk factors for fractures were disease duration, taken osteoporosis medications, age, higher BMI, history of previous bone fracture, corticosteroids use, seizures, cerebrovascular events, and SLICC/ACR-DI.

## 4. Discussion

In the current meta-analysis, as expected, the prevalence of low BMD at any site in SLE patients was high (45%), no matter in premenopausal patients (40%) or in postmenopausal patients (43%). The prevalence of osteopenia in all patients and premenopausal patients and postmenopausal patients was 38%, 42%, and 25%, while prevalence of osteoporosis was 13%, 9%, and 21%, respectively.

Higher prevalence of osteoporosis was shown at lumbar spine (13%) compared with the femur (6%) and hip (4%) in our study, which was consistent with some observational studies [2, 3, 17]. Lumbar spine was also reported to have high risk of fracture in SLE patients [9]. The mechanism for this discrepancy might be due to the widespread use of glucocorticoids for SLE therapy and the variable composition of each bone [18]. Trabecular bone is mainly affected by intensive glucocorticoid treatment [19], and the lumbar spine is mainly composed of trabecular bone. Low BMD and fracture of lumbar spine might have disastrous consequences. So, enough attention, timely examination, and effective intervention should be given to the bone density for SLE patients.

Several factors may explain the prevalence of low BMD in SLE patients.

First, SLE was a chronic autoimmune disease with multiorgan inflammation. The mean disease duration of SLE was 8.5 years and the mean SLEDAI score was 4.6 in our analysis. There were many disease-related variables playing a role in bone health. Increasing of tumor necrosis factor-*α* [20], interleukin-6 [21], and interleukin-1 [22] in the serum had an effect on the stimulation of bone resorption and inhibition of bone formation [23].

Second, corticosteroid therapy was frequent among SLE patients. From our literature search, the percentage of corticosteroid use was reported to be from 51.6% [24] to 100% [25, 26] (mean percentage: 78.8%; mean cumulative dose: 20.6 gram) and glucocorticoid use was reported to be associated with fractures [14, 27], osteoporosis [28, 29], and low BMD [30–32] in SLE patients. The mechanism might be that corticosteroid could inhibit the formation and function of osteoblast [33, 34], increase osteoclast, and inhibit absorption of calcium [35, 36], as well as affecting glucocorticoid induced leucine zipper proteins [37], 11*β*-hydroxysteroid dehy- drogenase type 1 [38], and other associated proteins to induce bone loss.

Third, postmenopausal status could lead to bone loss. In our study, postmenopausal patients (31.6% of all patients) showed higher prevalence of osteopenia, osteoporosis, and low BMD in almost all sites. Most of our SLE patients undergo early menopause, which may be due to SLE disease per se or its treatment with cytotoxic substances [39, 40]. Postmenopausal status was known as a risk factor of bone loss because of lacking estrogen, which affected the balance of bone formation and resorption metabolism through series of receptors and cytokines [41–45]. Postmenopausal status was reported to be associated with increased risk of low BMD (adjusted OR=3.32, 95% CI: 1.45-7.62) [46] and osteoporosis (adjusted OR=13.3, 95% CI: 1.6-111.1) [31]. Hence, it was necessary to place emphasis on the bone health for postmenopausal SLE patients.

Fourth, degeneration of bone increased with age. Older age was independently associated with bone loss in both general population [47] and SLE patients [2, 31]. Older age was also reported to be related to fracture [14, 48]. The overall average age of our included patients was 43.5 years and prevalence of osteoporosis increased with age. U-shaped associations between age and the prevalence of low BMD and osteopenia were found. For early-onset lupus and young patients, other factors, such as higher SLE disease index and inflammatory disease itself [49, 50], might influence the bone health. Similarly, we found that SLEDAI increased with the decreasing of age. In a word, age and SLE disease might affect the bone health at the same time.

Other risk factors might have effects on bone health in SLE patients. (1) The use of antimalarials might influence cytokines, lysosomal membranes, DNA, antigen processing, and other mechanisms that might lead to loss of bone. (2) The effect of body mass index (BMI) was still under debate. It was reported to be associated with increased risk of vertebral fracture (adjusted OR=1.17, 95% CI: 1.02-1.33) [48], but Lee et al. did not obtain the association with fracture (adjusted OR=1.01, 95% CI: 0.952-1.07) [12]. Meanwhile, higher BMI z score was associated with decreased risk of low BMD (adjusted OR=1.17, 95% CI: 1.02-1.33). More well-designed studies were needed to explore the association between BMI and bone loss. (3) Race, disease duration, the Systemic Lupus International Collaborating Clinics/American College of Rheumatology (SLICC/ACR) Damage Index, and many other risk factors were all reported to have associated with bone loss or fracture [27, 28, 31, 51].

We acknowledged some limitations of our analyses. Firstly, BMD measurements were performed in SLE patients with different disease duration. Secondly, the treatments for patients varied greatly. Thirdly, the differences regarding race, genetics, geographical locations, and lifestyle across the different population studied were not well considered. Fourthly, few studies reported the bone condition in male SLE patients, so we failed to extrapolate gender-related differences. Finally, the heterogeneity was high for most of the analyses.

## 5. Conclusions

SLE patients were at a great risk of developing low BMD, especially in lumbar spine and the postmenopausal patients. The risk factors that associated with low BMD might include low body weight, menopause duration, age, and disease-related factors.

## Figures and Tables

**Table 1:** Meta-analysis of the prevalence of osteopenia, osteoporosis, and low BMD in all SLE patients and postmenopausal and premenopausal patients.

Variable	*All patients*				*Premenopausal patients*				*Postmenopausal patients*			
	Number of studies	Sample size	Prevalence (95% CI)	I^2^	Number of studies	Sample size	Prevalence (95% CI)	I^2^	Number of studies	Sample size	Prevalence (95% CI)	I^2^
Osteopenia												
Spine	26	2951	35% (33, 38)	51.8%	4	264	29% (22, 37)	45.7%	4	189	37% (30, 44)	0.0%
Femur	15	1290	43% (38, 47)	66.0%	3	164	24% (17, 31)	0.0%	4	189	53% (38, 68)	74.8%
Total hip	15	2293	35% (27, 42)	92.7%	2	174	16% (11, 20)	0.0%	2	122	53% (26, 80)	88.0%
Any site	34	3319	38% (31, 45)	95.2%	8	502	42% (31, 52)	85.1%	3	234	25% (2, 48)	95.7%
Osteoporosis												
Spine	28	3317	13% (10, 15)	86.8%	9	512	13% (7, 19)	83.5%	4	189	27% (16, 37)	61.7%
Femur	15	1389	6% (5, 8)	8.0%	4	234	5% (2, 8)	4.2%	4	189	7% (2, 13)	54.3%
Total hip	15	2078	4% (3, 5)	63.7%	2	174	1% (-0, 3)	0.0%	2	122	12% (6, 18)	0.0%
Any site	42	29543	13% (11, 16)	90.8%	10	613	9% (5, 12)	73.0%	3	234	21% (11, 31)	74.0%
*Low BMD*												
Spine	29	3283	48% (43, 53)	89.2%	9	552	43% (32, 55)	88.9%	3	171	71% (56, 86)	79.2%
Femur	18	1602	47% (35, 59)	96.6%	5	257	39% (27, 51)	75.6%	4	189	61% (51, 71)	50.8%
Total hip	19	2686	36% (26, 46)	97.4%	4	301	16% (11, 20)	23.0%	2	122	65% (43, 83)	84.0%
Any site	40	5171	45% (38, 51)	96.6%	16	1179	40% (29, 51)	95.1%	2	153	43% (22, 63)	86.0%

**Table 2:** Risk factors for reduced bone mineral density in patients with SLE from the literature search.

Study (first author, year)	Country	Study design	Size	Female (%)	Mean age (y)	Outcome	Risk factor	Estimate (aOR, 95% CI)
LAKSHMINARAYANAN et al. 2001	USA	Prospective cohort	92	100	32.8	Low BMD	Menopause	3.32 (1.45, 7.62)
Yee et al. 2004	UK	Case control	242	95.5	39.9	Low BMD	Non-Afro-Caribbean ever taken prednisolone >10 mg/day	2.5 (1.2, 5.4) 2.1 (1.1, 4.2)
LEE et al. 2007	USA	Case control	298	100	42.1	Low hip BMD	African American race Age at SLE diagnosis BMI Drink caffeine SDI SLE renal disease Current use of GC Study center	1.54 (0.69, 3.46) 0.98 (0.95, 1.01) 0.90 (0.84, 0.96) 1.33 (0.53, 3.36) 1.30 (1.08, 1.57) 0.79 (0.33, 1.87) 1.48 (0.71, 3.09) 0.95 (0.42, 2.15)
LEE et al. 2007	USA	Case control	298	100	42.1	Low lumbar spine BMD	African American race Age at SLE diagnosis BMI/ kg/m2 Drink caffeine SDI SLE renal disease Current use of GC Study center	4.42 (2.19, 8.91) 0.96 (0.93, 0.99) 0.93 (0.89, 0.98) 0.34 (0.17, 0.72) 1.06 (0.89, 1.26) 1.50 (0.71, 3.16) 1.54 (0.81, 2.92) 0.71 (0.33, 1.53)
Furukawa et al. 2011	Japan	Case control	58	100	44.0	Low BMD	Number of deliveries Maximal dosage of >50 mg/day of oral GC	5.58 (1.31, 26.06) 0.25 (0.07, 0.91)
Lim et al. 2011	Canada	Retrospective cohort	80	82.5	14.2a	Low BMD	Higher BMI z score	0.35 (0.18, 0.69)
Bonfá et al. 2015	Brazil	Case control	365	100	32.8	Low BMD	Current GC use Osteoprotegerin 245 T>G	3.97 (1.51, 10.41) 2.14 (1.02, 4.50)
Seguro et al. 2015	Brazil	Prospective cohort	63	100	31.1	Low BMD	NPT1 Cumulative GC Mean GC Maximum GC	1.03 (1.01–1.05) 1.00 (1.00–1.00) 1.0 (0.95, 1.04) 0.98 (0.95, 1.01)
Cramarossa et al. 2016	Canada	Prospective cohort	286	88.8	38.0	Low BMD	Age Female Cumulative ACR criteria SDI excluding Osteoporosis Vitamin D use Calcium use Bisphosphonates use Immunosuppressives use Cumulative GC dose	1.06 (1.04, 1.08) ^*∗*^0.47 (0.23, 1.00) ^*∗*^0.80 (0.63, 1.02) ^*∗*^1.43 (1.12, 1.82) ^*∗*^1.63 (0.94, 2.80) ^*∗*^1.63 (0.94, 2.80) ^*∗*^1.72 (0.85, 3.47) ^*∗*^1.51 (0.90, 2.52) 1.04 (1.01, 1.07)
Lacassagne et al. 2007	Canada	Prospective cohort	64	76.6	14.3	Osteopenia	Cumulative GC dose	1.003 (1.001, 1.01)
Mak et al. 2011	Singapor	Case control	110	87	40.5	Osteopenia	Disease duration/month BMI/kg/m2 Cyclosporine use Cumulative GC dose FMD/% Carotid IMT/mm	1.031 (0.99, 1.073 0.78 (0.53, 1.16) 0.014 (0.00, 1.01) 1.080 (0.85, 1.38) 0.147 (0.02, 0.96) 0.000 (0.000)
SINIGAGLIA et al. 1999	Italy	Case control	84	100	30.5	Osteoporosis	Disease duration/year Prednisone/year-use	1.2 (1.07, 1.33) 1.16 (1.05, 1.29)
Banno et al. 2002	Japan	Case control	60	100	34.8	Osteoporosis	Cumulative GC intake	1.06 (1.01, 1.11)
Yee et al. 2004	UK	Case control	242	95.5	39.9	Osteoporosis	Menopause Age/year	13.3 (1.6, 111.1) 1.0 (1.0, 1.1)
Lacassagne et al. 2007	Canada	Prospective cohort	64	76.6	14.3	Osteoporosis	Disease duration/year lupus nephritis	1.60 (1.18, 2.18) 8.79 (0.96, 80.24)
Crosslin et al. 2011	USA	Case control	14829	90.5	47.3	Osteoporosis	Male	0.65 (0.43, 0.97)
Ajeganova et al. 2015	Sweden	Case control	222	89	48.7	Osteoporosis	Carotid plaque	1.78 (0.97, 3.24)

Note: OR, odds ratio; USA, United States of America; BMD, bone mineral density; UK, the United Kingdom of Great Britain and Northern Ireland; BMI, Body mass index; SLICC/ACR-DI, the Systemic Lupus International Collaborating Clinics/American College of Rheumatology-Damage Index; GC, glucocorticoid; NPT1, N-terminal propeptide of type 1 collagen; FMD, flow-mediated dilatation. Hint: ^a^median; ^*∗*^aRR: adjusted risk ratio.

## Data Availability

All relevant data are within the paper and its Supplementary Materials files.

## Supplementary Materials

Supplementary 1 S1 file: PRISMA 2009 Checklist.

Supplementary 2 S2 file: Search strategy to identify studies with data on prevalence of reduced bone density in SLE patients.

Supplementary 3 S3 file: References of studies included in the meta-analysis.

Supplementary 4 S4 file: Characteristics of included studies.
