# Supplementary material for: Prevalence and Risk Factors of Reduced Bone Mineral Density in Systemic Lupus Erythematosus Patients: A Meta-Analysis
Source: Biomed Res Int. 2019 Feb 20;2019:3731648. doi: 10.1155/2019/3731648 (PMC6402203; doi:10.1155/2019/3731648)
Supplement: Supplementary 2 — S2 file: Search strategy to identify studies with data on prevalence of reduced bone density in SLE patients. [file 3731648.f2.docx]

**Supplementary 2. S2 file. Search strategy to identify studies with data on prevalence of reduced bone density in SLE patients.**

**On Pubmed**

Search (((((((bone mineral density) OR bone density) OR Osteoporosis) OR Osteopenia) OR fracture)) AND (((risk factors) OR outcomes) OR Prevalence)) AND ((Systemic Lupus Erythematosus) OR SLE)

**On Web of Science^TM^**

Search (((((((bone mineral density) OR bone density) OR Osteoporosis) OR Osteopenia) OR fracture)) AND (((risk factors) OR outcomes) OR Prevalence)) AND ((Systemic Lupus Erythematosus) OR SLE)

**On Cochrance**

#1: bone mineral density or bone density or Osteoporosis or Osteopenia or fracture (Word variations have been searched)

#2: risk factors or outcomes or Prevalence (Word variations have been searched)

#3: Systemic Lupus Erythematosus or SLE (Word variations have been searched)

#4: #1 and #2 and #3

Choose # 4

**On EMBASE**

1. SLE

2. Systemic Lupus Erythematosus

3. risk factors

4. outcomes

5. Prevalence

6. bone mineral density

7. bone density

8. Osteoporosis

9. Osteopenia

10. fracture

11. 1 or 2

12. 3 or 4 or 5

13. 6 or 7 or 8 or 9 or 10

14. 11 and 12 and 13

CHOOSE 14
