# Supplementary material for: Prevalence and Risk Factors of Reduced Bone Mineral Density in Systemic Lupus Erythematosus Patients: A Meta-Analysis
Source: Biomed Res Int. 2019 Feb 20;2019:3731648. doi: 10.1155/2019/3731648 (PMC6402203; doi:10.1155/2019/3731648)
Supplement: Supplementary 3 — S3 file: References of studies included in the meta-analysis. [file 3731648.f3.docx]

**Supplementary 3. S3 file. References of studies included in the meta-analysis.**

[[1-71](#_ENREF_1)]

1. Guo Q, Fan P, Luo J, Wu S, Sun H, He L, Zhou B: **Assessment of bone mineral density and bone metabolism in young male adults recently diagnosed with systemic lupus erythematosus in China**. *Lupus* 2016.

2. La Montagna G, Baruffo A, Maja L, Matrone C: **Bone mineral density in young females with systemic lupus erythematosus. Influence of glucocorticoids. [Italian] Densita minerale ossea in giovani donne affette da lupus eritematoso sistemico. Influenza dei glucocorticoidi**. *Reumatismo* 1997, **49**(2):85-90.

3. Sinigaglia L, Varenna M, Binelli L, Zucchi F, Ghiringhelli D, Gallazzi M, Limonta M, Zeni S, Fantini F: **Determinants of bone mass in systemic lupus erythematosus: A cross sectional study on premenopausal women**. *Journal of Rheumatology* 1999, **26**(6):1280-1284.

4. So MY, Mok CC, Ma KM, Kwok A, Leung PC, Wong SN: **Frequency of low bone mineral density and its associated factors in patients with juvenile systemic lupus erythemat**. *Hong Kong Journal of Paediatrics* 2011, **16**(2):77-84.

5. Zurek M, Horak P, Pospisil Z, Kraina T, Kusa L, Scudla V: **The evaluation of bone mineral density and risk factors of osteoporosis in patients with diffuse connective tissue diseases. [Czech] Hodnoceni denzity kostniho mineralu a rizikovych faktoru osteoporozy u nemocnych se systemovymi chorobami pojiva**. *Osteologicky Bulletin* 2003, **8**(4):125-130.

6. Zurek M, Horak P, Smrzova A, Skacelova M: **Longitudinal study of bone mineral density changes in patients with systemic lupus erythematosus. [Czech] Longitudinalni sledovani zmen denzity kostniho mineralu u nemocnych se systemovym lupus erytematodes**. *Osteologicky Bulletin* 2010, **15**(1):9-17.

7. Bhattoa HP, Kiss E, Bettembuk P, Balogh A: **Bone mineral density, biochemical markers of bone turnover, and hormonal status in men with systemic lupus erythematosus**. *Rheumatology international* 2001, **21**(3):97-102.

8. Abdwani R, Abdulla E, Yaroubi S, Bererhi H, Al-Zakwani I: **Bone mineral density in juvenile onset systemic lupus erythematosus**. *Indian pediatrics* 2015, **52**(1):38-40.

9. Uaratanawong S, Deesomchoke U, Lertmaharit S, Uaratanawong S: **Bone mineral density in premenopausal women with systemic lupus erythematosus**. *The Journal of rheumatology* 2003, **30**(11):2365-2368.

10. Gilboe IM, Kvien TK, Haugeberg G, Husby G: **Bone mineral density in systemic lupus erythematosus: comparison with rheumatoid arthritis and healthy controls**. *Annals of the rheumatic diseases* 2000, **59**(2):110-115.

11. Bhattoa HP, Bettembuk P, Balogh A, Szegedi G, Kiss E: **Bone mineral density in women with systemic lupus erythematosus**. *Clinical rheumatology* 2002, **21**(2):135-141.

12. Mendoza-Pinto C, Garcia-Carrasco M, Jimenez-Hernandez M, Sanchez-Perez R, Escarcega RO, Nava-Zavala A, Munguia-Realpozo P, Lopez-Colombo A, Jara LJ, Cervera R: **Carotid atherosclerosis is not associated with lower bone mineral density and vertebral fractures in patients with systemic lupus erythematosus**. *Lupus* 2015, **24**(1):25-31.

13. Lakshminarayanan S, Walsh S, Mohanraj M, Rothfield N: **Factors associated with low bone mineral density in female patients with systemic lupus erythematosus**. *The Journal of rheumatology* 2001, **28**(1):102-108.

14. Casella CB, Seguro LP, Takayama L, Medeiros D, Bonfa E, Pereira RM: **Juvenile onset systemic lupus erythematosus: a possible role for vitamin D in disease status and bone health**. *Lupus* 2012, **21**(12):1335-1342.

15. Li EK, Tam LS, Young RP, Ko GT, Li M, Lau EM: **Loss of bone mineral density in Chinese pre-menopausal women with systemic lupus erythematosus treated with corticosteroids**. *British journal of rheumatology* 1998, **37**(4):405-410.

16. Kalla AA, Fataar AB, Jessop SJ, Bewerunge L: **Loss of trabecular bone mineral density in systemic lupus erythematosus**. *Arthritis and rheumatism* 1993, **36**(12):1726-1734.

17. Pineau CA, Urowitz MB, Fortin PJ, Ibanez D, Gladman DD: **Osteoporosis in systemic lupus erythematosus: factors associated with referral for bone mineral density studies, prevalence of osteoporosis and factors associated with reduced bone density**. *Lupus* 2004, **13**(6):436-441.

18. Cramarossa G, Urowitz MB, Su J, Gladman D, Touma Z: **Prevalence and associated factors of low bone mass in adults with systemic lupus erythematosus**. *Lupus* 2016.

19. Carli L, Tani C, Spera V, Vagelli R, Vagnani S, Mazzantini M, Di Munno O, Mosca M: **Risk factors for osteoporosis and fragility fractures in patients with systemic lupus erythematosus**. *Lupus science & medicine* 2016, **3**(1):e000098.

20. Lee C, Almagor O, Dunlop DD, Manzi S, Spies S, Ramsey-Goldman R: **Self-reported fractures and associated factors in women with systemic lupus erythematosus**. *The Journal of rheumatology* 2007, **34**(10):2018-2023.

21. Korczowska I, Olewicz-Gawlik A, Hrycaj P, Lacki J: **The effect of long-term glucocorticoids on bone metabolism in systemic lupus erythematosus patients: the prevalence of its anti-inflammatory action upon bone resorption**. *The Yale journal of biology and medicine* 2003, **76**(2):45-54.

22. Toloza SM, Cole DE, Gladman DD, Ibanez D, Urowitz MB: **Vitamin D insufficiency in a large female SLE cohort**. *Lupus* 2010, **19**(1):13-19.

23. Zhu TY, Griffith JF, Au SK, Tang XL, Kwok AW, Leung PC, Li EK, Tam LS: **Incidence of and risk factors for non-vertebral and vertebral fracture in female Chinese patients with systemic lupus erythematosus: a five-year cohort study**. *Lupus* 2014, **23**(9):854-861.

24. Ajeganova S, Gustafsson T, Jogestrand T, Frostegard J, Hafstrom I: **Bone mineral density and carotid atherosclerosis in systemic lupus erythematosus: a controlled cross-sectional study**. *Arthritis research & therapy* 2015, **17**:84.

25. Almehed K, Forsblad d'Elia H, Kvist G, Ohlsson C, Carlsten H: **Prevalence and risk factors of osteoporosis in female SLE patients-extended report**. *Rheumatology (Oxford, England)* 2007, **46**(7):1185-1190.

26. Becker A, Fischer R, Scherbaum WA, Schneider M: **Osteoporosis screening in systemic lupus erythematosus: impact of disease duration and organ damage**. *Lupus* 2001, **10**(11):809-814.

27. Bertoli AM, Alarcon GS, Calvo-Alen J, Fernandez M, Vila LM, Reveille JD, Group LS: **Systemic lupus erythematosus in a multiethnic US cohort. XXXIII. Clinical [corrected] features, course, and outcome in patients with late-onset disease**. *Arthritis and rheumatism* 2006, **54**(5):1580-1587.

28. Bonfa AC, Seguro LP, Caparbo V, Bonfa E, Pereira RM: **RANKL and OPG gene polymorphisms: associations with vertebral fractures and bone mineral density in premenopausal systemic lupus erythematosus**. *Osteoporosis international : a journal established as result of cooperation between the European Foundation for Osteoporosis and the National Osteoporosis Foundation of the USA* 2015, **26**(5):1563-1571.

29. Borba VZ, Matos PG, da Silva Viana PR, Fernandes A, Sato EI, Lazaretti-Castro M: **High prevalence of vertebral deformity in premenopausal systemic lupus erythematosus patients**. *Lupus* 2005, **14**(7):529-533.

30. Boyanov M, Robeva R, Popivanov P: **Bone mineral density changes in women with systemic lupus erythematosus**. *Clinical rheumatology* 2003, **22**(4-5):318-323.

31. Bultink IE, Lems WF, Kostense PJ, Dijkmans BA, Voskuyl AE: **Prevalence of and risk factors for low bone mineral density and vertebral fractures in patients with systemic lupus erythematosus**. *Arthritis and rheumatism* 2005, **52**(7):2044-2050.

32. Caetano M, Terreri MT, Ortiz T, Pinheiro M, Souza F, Sarni R: **Bone mineral density reduction in adolescents with systemic erythematosus lupus: association with lack of vitamin D supplementation**. *Clinical rheumatology* 2015, **34**(12):2065-2070.

33. Cervera R, Khamashta MA, Font J, Sebastiani GD, Gil A, Lavilla P, Mejia JC, Aydintug AO, Chwalinska-Sadowska H, de Ramon E *et al*: **Morbidity and mortality in systemic lupus erythematosus during a 10-year period: a comparison of early and late manifestations in a cohort of 1,000 patients**. *Medicine* 2003, **82**(5):299-308.

34. Chan PC, Yu CH, Yeh KW, Horng JT, Huang JL: **Comorbidities of pediatric systemic lupus erythematosus: A 6-year nationwide population-based study**. *Journal of microbiology, immunology, and infection = Wei mian yu gan ran za zhi* 2016, **49**(2):257-263.

35. Chong HC, Chee SS, Goh EM, Chow SK, Yeap SS: **Dietary calcium and bone mineral density in premenopausal women with systemic lupus erythematosus**. *Clinical rheumatology* 2007, **26**(2):182-185.

36. Coimbra Ibsen B, Costallat Lilian Tereza L: **Bone mineral density in systemic lupus erythematosus and its relation to age at disease onset, plasmatic estradiol and immunosuppressive therapy**. *Joint Bone Spine* 2003, **70**(1):40-45.

37. Compeyrot-Lacassagne S, Tyrrell PN, Atenafu E, Doria AS, Stephens D, Gilday D, Silverman ED: **Prevalence and etiology of low bone mineral density in juvenile systemic lupus erythematosus**. *Arthritis and rheumatism* 2007, **56**(6):1966-1973.

38. Crosslin KL, Wiginton KL: **Sex differences in disease severity among patients with systemic lupus erythematosus**. *Gender medicine* 2011, **8**(6):365-371.

39. Demas KL, Keenan BT, Solomon DH, Yazdany J, Costenbader KH: **Osteoporosis and cardiovascular disease care in systemic lupus erythematosus according to new quality indicators**. *Seminars in arthritis and rheumatism* 2010, **40**(3):193-200.

40. Fischer-Betz R, Wessel E, Richter J, Winkler-Rohlfing B, Willers R, Schneider M: **[Lupus in Germany: analysis within the German lupus self-help organization (LULA)]**. *Zeitschrift fur Rheumatologie* 2005, **64**(2):111-122.

41. Furukawa M, Kiyohara C, Tsukamoto H, Mitoma H, Kimoto Y, Uchino A, Nakagawa M, Oryoji K, Shimoda T, Akashi K *et al*: **Prevalence of and risk factors for low bone mineral density in Japanese female patients with systemic lupus erythematosus**. *Rheumatology international* 2011, **31**(3):365-376.

42. Jacobs J, Korswagen LA, Schilder AM, van Tuyl LH, Dijkmans BA, Lems WF, Voskuyl AE, Bultink IE: **Six-year follow-up study of bone mineral density in patients with systemic lupus erythematosus**. *Osteoporosis international : a journal established as result of cooperation between the European Foundation for Osteoporosis and the National Osteoporosis Foundation of the USA* 2013, **24**(6):1827-1833.

43. Lee C, Almagor O, Dunlop DD, Manzi S, Spies S, Chadha AB, Ramsey-Goldman R: **Disease damage and low bone mineral density: an analysis of women with systemic lupus erythematosus ever and never receiving corticosteroids**. *Rheumatology (Oxford, England)* 2006, **45**(1):53-60.

44. Lee JJ, Aghdassi E, Cheung AM, Morrison S, Cymet A, Peeva V, Neville C, Hewitt S, DaCosta D, Pineau C *et al*: **Ten-year absolute fracture risk and hip bone strength in Canadian women with systemic lupus erythematosus**. *The Journal of rheumatology* 2012, **39**(7):1378-1384.

45. Li EK, Tam LS, Griffith JF, Zhu TY, Li TK, Li M, Wong KC, Chan M, Lam CW, Chu FS *et al*: **High prevalence of asymptomatic vertebral fractures in Chinese women with systemic lupus erythematosus**. *The Journal of rheumatology* 2009, **36**(8):1646-1652.

46. Li EK, Zhu TY, Tam LS, Hung VW, Griffith JF, Li TK, Li M, Wong KC, Leung PC, Kwok AW *et al*: **Bone microarchitecture assessment by high-resolution peripheral quantitative computed tomography in patients with systemic lupus erythematosus taking corticosteroids**. *The Journal of rheumatology* 2010, **37**(7):1473-1479.

47. Lilleby V, Lien G, Frey Froslie K, Haugen M, Flato B, Forre O: **Frequency of osteopenia in children and young adults with childhood-onset systemic lupus erythematosus**. *Arthritis and rheumatism* 2005, **52**(7):2051-2059.

48. Lim LS, Benseler SM, Tyrrell PN, Harvey E, Herbert D, Charron M, Silverman ED: **Predicting longitudinal trajectory of bone mineral density in paediatric systemic lupus erythematosus patients**. *Annals of the rheumatic diseases* 2012, **71**(10):1686-1691.

49. Lim SH, Benseler SM, Tyrrell PN, Charron M, Harvey E, Hebert D, Silverman ED: **Low bone mineral density is present in newly diagnosed paediatric systemic lupus erythematosus patients**. *Annals of the rheumatic diseases* 2011, **70**(11):1991-1994.

50. Mak A, Lim JQ, Liu Y, Cheak AA, Ho RC: **Significantly higher estimated 10-year probability of fracture in lupus patients with bone mineral density comparable to that of healthy individuals**. *Rheumatology international* 2013, **33**(2):299-307.

51. Mendoza Pinto C, Garcia Carrasco M, Etchegaray Morales I, Jimenez Hernandez M, Mendez Martinez S, Jimenez Hernandez C, Briones Rojas R, Ramos Alvarez G, Rodriguez Gallegos A, Montiel Jarquin A *et al*: **Bone mineral density in systemic lupus erythematosus women one year after rituximab therapy**. *Lupus* 2013, **22**(11):1128-1134.

52. Mendoza-Pinto C, Garcia-Carrasco M, Sandoval-Cruz H, Escarcega RO, Jimenez-Hernandez M, Etchegaray-Morales I, Soto-Vega E, Munoz-Guarneros M, Lopez-Colombo A, Deleze-Hinojosa M *et al*: **Risks factors for low bone mineral density in pre-menopausal Mexican women with systemic lupus erythematosus**. *Clinical rheumatology* 2009, **28**(1):65-70.

53. Mendoza-Pinto C, Garcia-Carrasco M, Sandoval-Cruz H, Munoz-Guarneros M, Escarcega RO, Jimenez-Hernandez M, Munguia-Realpozo P, Sandoval-Cruz M, Deleze-Hinojosa M, Lopez-Colombo A *et al*: **Risk factors of vertebral fractures in women with systemic lupus erythematosus**. *Clinical rheumatology* 2009, **28**(5):579-585.

54. Mok CC, Mak A, Ma KM: **Bone mineral density in postmenopausal Chinese patients with systemic lupus erythematosus**. *Lupus* 2005, **14**(2):106-112.

55. Mok CC, Wong SN, Ma KM: **Childhood-onset disease carries a higher risk of low bone mineral density in an adult population of systemic lupus erythematosus**. *Rheumatology (Oxford, England)* 2012, **51**(3):468-475.

56. Molina MJ, Mayor AM, Franco AE, Morell CA, Lopez MA, Vila LM: **Prevalence of systemic lupus erythematosus and associated comorbidities in Puerto Rico**. *Journal of clinical rheumatology : practical reports on rheumatic & musculoskeletal diseases* 2007, **13**(4):202-204.

57. Peracchi OAB, Terreri MTRA, Munekata RV, Len CA, Sarni ROS, Lazaretti-Castro M, Hilário MOE: **Low serum concentrations of 25-hydroxyvitamin D in children and adolescents with systemic lupus erythematosus**. *Brazilian Journal of Medical and Biological Research* 2014, **47**(8):721-726.

58. Rees F, Doherty M, Grainge M, Lanyon P, Davenport G, Zhang W: **Burden of Comorbidity in Systemic Lupus Erythematosus in the UK, 1999-2012**. *Arthritis care & research* 2016, **68**(6):819-827.

59. Regio P, Bonfa E, Takayama L, Pereira R: **The influence of lean mass in trabecular and cortical bone in juvenile onset systemic lupus erythematosus**. *Lupus* 2008, **17**(9):787-792.

60. Ribeiro GG, Bonfa E, Sasdeli Neto R, Abe J, Caparbo VF, Borba EF, Lopes JB, Gebrim E, Pereira RM: **Premature coronary artery calcification is associated with disease duration and bone mineral density in young female systemic lupus erythematosus patients**. *Lupus* 2010, **19**(1):27-33.

61. Salman-Monte TC, Torrente-Segarra V, Almirall M, Corzo P, Mojal S, Carbonell-Abello J: **Prevalence and predictors of vitamin D insufficiency in supplemented and non-supplemented women with systemic lupus erythematosus in the Mediterranean region**. *Rheumatology international* 2016, **36**(7):975-985.

62. Salman-Monte TC, Torrente-Segarra V, Munoz-Ortego J, Mojal S, Carbonell-Abello J: **Prevalence and predictors of low bone density and fragility fractures in women with systemic lupus erythematosus in a Mediterranean region**. *Rheumatology international* 2015, **35**(3):509-515.

63. Seguro LP, Casella CB, Caparbo VF, Oliveira RM, Bonfa A, Bonfa E, Pereira RM: **Lower P1NP serum levels: a predictive marker of bone loss after 1 year follow-up in premenopausal systemic lupus erythematosus patients**. *Osteoporosis international : a journal established as result of cooperation between the European Foundation for Osteoporosis and the National Osteoporosis Foundation of the USA* 2015, **26**(2):459-467.

64. Shaharir SS, Hussein H, Rajalingham S, Mohamed Said MS, Abdul Gafor AH, Mohd R, Mustafar R: **Damage in the Multiethnic Malaysian Systemic Lupus Erythematosus (SLE) Cohort: Comparison with Other Cohorts Worldwide**. *PloS one* 2016, **11**(11):e0166270.

65. Souto MI, Coelho A, Guo C, Mendonca LM, Pinheiro MF, Papi JA, Farias ML: **The prevalence of low bone mineral density in Brazilian patients with systemic lupus erythematosus and its relationship with the disease damage index and other associated factors**. *Journal of clinical densitometry : the official journal of the International Society for Clinical Densitometry* 2012, **15**(3):320-327.

66. Sun YN, Feng XY, He L, Zeng LX, Hao ZM, Lv XH, Pu D: **Prevalence and possible risk factors of low bone mineral density in untreated female patients with systemic lupus erythematosus**. *BioMed research international* 2015, **2015**:510514.

67. Tang XL, Qin L, Kwok AW, Zhu TY, Kun EW, Hung VW, Griffith JF, Leung PC, Li EK, Tam LS: **Alterations of bone geometry, density, microarchitecture, and biomechanical properties in systemic lupus erythematosus on long-term glucocorticoid: a case-control study using HR-pQCT**. *Osteoporosis international : a journal established as result of cooperation between the European Foundation for Osteoporosis and the National Osteoporosis Foundation of the USA* 2013, **24**(6):1817-1826.

68. Tang XL, Zhu TY, Hung VW, Qin L, Wong CK, Kun EW, Tam LS, Li EK: **Increased organ damage associated with deterioration in volumetric bone density and bone microarchitecture in patients with systemic lupus erythematosus on longterm glucocorticoid therapy**. *The Journal of rheumatology* 2012, **39**(10):1955-1963.

69. Tomic-Lucic A, Petrovic R, Radak-Perovic M, Milovanovic D, Milovanovic J, Zivanovic S, Pantovic S, Veselinovic M: **Late-onset systemic lupus erythematosus: clinical features, course, and prognosis**. *Clinical rheumatology* 2013, **32**(7):1053-1058.

70. Yeap SS, Fauzi AR, Kong NC, Halim AG, Soehardy Z, Rahimah S, Chow SK, Goh EM: **Influences on bone mineral density in Malaysian premenopausal systemic lupus erythematosus patients on corticosteroids**. *Lupus* 2009, **18**(2):178-181.

71. Yee CS, Crabtree N, Skan J, Amft N, Bowman S, Situnayake D, Gordon C: **Prevalence and predictors of fragility fractures in systemic lupus erythematosus**. *Annals of the rheumatic diseases* 2005, **64**(1):111-113.
