# Supplementary material for: Prevalence and Risk Factors of Reduced Bone Mineral Density in Systemic Lupus Erythematosus Patients: A Meta-Analysis
Source: Biomed Res Int. 2019 Feb 20;2019:3731648. doi: 10.1155/2019/3731648 (PMC6402203; doi:10.1155/2019/3731648)
Supplement: Supplementary 4 — S4 file: Characteristics of included studies. [file 3731648.f4.docx]

**Supplementary 4. S4 file. Characteristics of included studies.**

| Study | Sample size | Mean age,  y | Female,% | SLEDAI | SLICC/SDI | BMI, kg/m^2^ | Postmen-opausal, % | GCs ever user, % | Cumulative GCs dose, g | Age at disease onset, y | Disease duration, y | site of body | Bone disease |
| --- | --- | --- | --- | --- | --- | --- | --- | --- | --- | --- | --- | --- | --- |
| ABDWANI  et al. 2014 | 27 | 11.0 | 74.0 | 16.0 | - | 16 | 0 | 100 | 13.0 | 6.0 | 4.0 | any site | low BMD, osteoporosis, osteopenia |
| Ajeganova  et al. 2015 | 111 | 48.7 | 89.0 | 2.0 | 1 | - | 51 | 60.4 | 11.4 | - | 9.0 | any site, lumbar spine, total hip, femoral neck | low BMD |
| Almehed  et al. 2007 | 162 | 47.0 | 100 | 5.0 | 2.0 | 24.2 | 55 | 85.0 | 18.8 | - | 11.0 | any site, Lumbal spine, total hip, femoral neck | low BMD, osteopenia, osteoporosis |
| Becker  et al. 2001 | 64 | 34.5 | 52.0 | 9.0* | 0* | - | - | 97.0 | - | - | 7.7 | any site, lumbar spine，femoral neck | low BMD, osteoporosis, osteopenia |
| Bertoli  et al. 2006 | 217 | 42.2 | 85.0 | 9.2 | 2.0 | - | - | - | - | - | 5.2 | any site | osteoporosis |
| Betz  et al. 2005 | 1033 | - | 92.2 | - | - | - | - | 67.9 | - | - | 9.9 | any site | osteoporosis |
| Bhattoa  et al. 2001 | 23 | 45.6 | 0 | 2.1 | 3.9 | 25.3 | 0 | 91.0 | 33.4 | - | 10.1 | lumbar spine， femoral neck，left forearm | low BMD, osteoporosis, osteopenia |
| Bhattoa  et al. 2002 | 79 | 49.0 | 100 | 4.0 | - | 26.0 | 62.0 | 89.7 | 13.9 | - | 9.0 | lumbar spine， femoral neck | low BMD, osteoporosis, osteopenia |
| Bonfá  et al. 2015 | 211 | 33.3 | 100 | 2.4 | 0.7 | 27.0 | 0 | 75.4 | 28.6 | - | 7.6 | any site | low BMD |
| Borba  et al. 2005 | 70 | 31.3 | 100 | - | - | 25.2 | -- | - | - | 25.2 | 6.3 | any site | low BMD, osteoporosis, osteopenia |
| Boyanov  et al. 2003 | 32 | 43.2 | 100 | - | - | 24.3 | - | - | 34.4 | - | 13.4 | any site | low BMD, osteoporosis, osteopenia |
| Bultink  et al. 2005 | 107 | 41.0 | 93 | 4.9 | 1.4 | 25.0 | 28.0 | 81.0 | - | - | 6.9 | any site, lumbar spine，total hip | low BMD, osteoporosis, osteopenia |
| Caetano  et al. 2015 | 35 | 15.4 | 100 | 4.0 | - | - | 0 | - | 13.8* | - | 3.7 | any site | low BMD |
| Carli  et al. 2016 | 186 | 46.4 | 94.1 | - | - | 23.6 | 33.1 | 100 | 34.9 | - | 14.9 | any site, spine, femoral | low BMD, osteoporosis, osteopenia |
| Casella  et al. 2012 | 57 | 19.5 | 80.7 | 2.3 | 0.3 | 23.1 | 0 | - | - | - | 7.4 | any site | low BMD |
| Cervera  et al. 2003 | 1000 | 37.0 | 90.8 | - | - | - | - | 72.5 | - | - | - | any site | osteoporosis |
| Chan  et al.2014 | 904 | - | 85.6 | - | - | - | - | - | - | - | - | any site | osteoporosis |
| Chong  et al. 2007 | 60 | 33.7 | 100 | 10.0* | - | - | 0 | 100 | 17.2* | - | 25.3 | any site | low BMD, osteoporosis, osteopenia |
| Coimbra  et al. 2003 | 60 | 32.8 | 100 | - | - | 25.1 | 0 | 98.3 | 28.8 | - | - | lumbar spine, Proximal femur | low BMD, osteoporosis, osteopenia |
| Cramarossa  et al. 2016 | 286 | 38.0 | 88.8 | 7.4 | 0.6 | - | - | 73.1 | 0.01 | - | 2.2 | any site | low BMD, osteoporosis, osteopenia |
| Crosslin  et al. 2011 | 14829 |  | 90.5 | - | - | - | - | - | - | - | - | any site | osteoporosis |
| Demas  et al. 2010 | 67 | 46.3 | 94.0 | - | - | 26.9 | - | - | - | - | 15.3 | any site | low BMD, osteoporosis, osteopenia |
| Furukawa  et al. 2011 | 58 | 44.0 | 100 | 7.0 | - | 22.4 | 33.3 | 96.6 | - | - | 16.5 | any site, lumbar spine，total hip | low BMD, osteoporosis, osteopenia |
| Gilboe  et al. 2010 | 75 | 45.0 | 88.0 | 6.9 | 2.1 | 23.5 | 57.3 | 85.0 | 21.8 | - | 8.1 | lumbar spine, femoral neck, total hip | low BMD, osteoporosis, osteopenia |
| Guo  et al. 2016 | 60 | 25.8 | 0 | 12.2 | - | 21.2 | 0 | - | - | - | - | lumbar spine, total hip | low BMD, osteoporosis, osteopenia |
| Jacobs  et al. 2012 | 126 | 39.0 | 89.7 | 4.0* | 0.5 | 24.0 | 18.3 | 51.6 | - | - | - | any site, lumbar spine, hip | low BMD, osteoporosis, osteopenia |
| KALLA  et al. 1993 | 46 | 31.0 | 100 | - | - | - | - | - | - | - | 6.3 | femoral | low BMD |
| Korczowska  et al. 2003 | 38 | 46.4 | 100 | - | - | - | 57.9 | 100 | 25.3 | - | 9.0 | any site | osteoporosis |
| Lacassagne  et al. 2007 | 64 | 14.3 | 76.6 | 4.6 | - | 22.2 | 0 | 96.8 | - | 11.4 | 2.9 | Lumbar spine, total hip | low BMD, osteoporosis, osteopenia |
| LAKSHMINARAYANAN et al. 2001 | 92 | 45.9 | 100 | 2.4 | 1.2 | - | 52.2 | 97.8 | 4.1 | 32.8 | 14.5 | any site, lumbar spine，Femoral Hip | low BMD, osteoporosis, osteopenia |
| Lee  et al. 2006 | 307 | 41.7 | 100 | - | 1.3 | 26.9 | 35.0 | 77.5 | - | 32.7 | 8.5 | total hip, lumbar spine | low BMD, osteoporosis, osteopenia |
| Lee  et al. 2007 | 304 | 41.7 | 100 | - | 1.3 | 26.9 | 38.1 | 78.9 | - | 32.5 | 8.7 | any site | low BMD |
| LEE  et al. 2012 | 271 | 43.8 | 100 | - | 1.2 | 26.3 | - | 69.2 | - | 32.2 | 11.6 | any site, total spine，Femoral neck, total hip | low BMD, osteoporosis, osteopenia |
| LI  et al. 1998 | 52 | 34.1 | 100 | 4.3 | - | 20.7 | 0 | 100 | 10.5 | - | 6.4 | any site | low BMD, osteoporosis, osteopenia |
| LI  et al. 2009 | 152 | 47.9 | 100 | 1.8 | 1 | 22.4 | 68.4 | 91.4 | - | - | - | any site, lumbar spine，total hip | low BMD, osteoporosis, osteopenia |
| LI  et al. 2010 | 59 | 46.9 | 100 | 2.1 | 1.1 | 22.8 | 59.0 | - | 31.0 | - | 12.9 | any site | low BMD, osteoporosis, osteopenia |
| Lilleby  et al. 2005 | 70 | 26.4 | 76.0 | 3.0 | 1.3 | 24.2 | 0 | 93.0 | 19.3 | 12.5 | 10.8 | femoral neck, lumbar spine | osteoporosis |
| Lim  et al. 2011 | 80 | 13.1 | 82.5 | - | - | 19.8 | 0 | - | - | 13.1* | - | any site | low BMD |
| Lim  et al. 2012 | 68 | 13.1 | 84.0 | - | - | 20.3 | 0 | - | - | 13.1* | - | lumbar spine | low BMD, osteoporosis, osteopenia |
| Lucic  et al. 2013 | 30 | 33.8 | 93.3 | - | - | - | - | - | - | - | 3.5 | any site | osteoporosis |
| Mak  et al. 2013 | 45 | 50.1 | 82.0 | 5.2 | 0.4 | 23.1 | 65.0 | 87.0 | - | - | 6.2 | any site | osteoporosis |
| Mok  et al. 2005 | 34 | 52.9 | 100 | 1* | 1 | 23.7 | 100 | 56.0 | 11.3 | - | 6.3 | lumbar spine, femoral neck, total hip | low BMD, osteoporosis, osteopenia |
| Mok  et al. 2012 | 395 | 40.3 | 94.0 | - | - | 22.2 | 32.9 | - | - | 32.5 | 7.8 | lumbar spine, hip, femoral neck | low BMD, osteoporosis, osteopenia |
| Molina  et al. 2007 | 877 | 42.0 | 92.6 | - | - | - | 56.7 | - | - | - | 3.0 | any site | low BMD |
| Montagna  et al. 1997 | 38 | 29.4 | 100 | - | - | - | 0 | 100 | - | 8.7 | - | any site, proximal forearm | low BMD, osteoporosis, osteopenia |
| Monte  et al. 2015 | 66 | 53.2 | 100 | 0 | 0 | - | 56.7 | 80.5 | - | - | 3.0 | lumbar spine, femoral neck, total hip | low BMD, osteoporosis, osteopenia |
| Monte  et al. 2016 | 102 | 52.4 | 100 | 3.8 | - | 24.8 | 59.8 | - | - | - | - | lumbar spine, femoral neck, total hip | low BMD, osteoporosis, osteopenia |
| Peracchi  et al. 2014 | 30 | 13.7 | 83.3 | - | - | - | 0 | 53.0 | 37.0 | 10.5 | 3.4 | any site | low BMD |
| Pineau  et al. 2004 | 205 | 45.2 | 100 | 6.1 | - | - | 42.4 | 89.6 | - | - | 13.8 | any site | low BMD, osteoporosis, osteopenia |
| Pinto  et al. 2009 | 210 | 43.3 | 100 | 3.4 | 1.1 | 27.3 | 49.4 | - | 19.2 | - | 8.2 | any site, Lumbal spine, total hip | low BMD, osteoporosis, osteopenia |
| Pinto  et al. 2009 (2) | 100 | 32.8 | 100 | 3.6 | 1.1 | 26.5 | 0 | 99.0 | 13.6* | - | 4* | any site, Lumbal spine, total hip | low BMD, osteoporosis, osteopenia |
| Pinto  et al. 2013 | 76 | 41.3 | 100 | 2.3 | 0.6 | 27.3 | 46.1 | 97.4 | 23.5 | - | 9.5 | any site | low BMD, osteoporosis, osteopenia |
| Pinto  et al. 2015 | 65 | - | 100 | - | - | - | 100 | - | - | - | - | any site | low BMD, osteoporosis, osteopenia |
| Rees  et al. 2015 | 7732 | 48.1 | 85.8 | - | - | - | - | - | - | - | 2.4 | any site | osteoporosis |
| Regio  et al. 2008 | 31 | 17.7 | - | 3.7 | 0.5 | - | - | - | 24.9 | - | 5.1 | any site | low BMD |
| Ribeiro  et al. 2010 | 94 | 33.6 | 100 | 2.8 | 1.6 | 26.7 | 13.8 | - | - | - | 13.0 | any site | osteoporosis |
| Seguro et al. 2015 | 63 | 31.1 | 100 | 4.4 | 0.7 | 26.1 | 0 | - | 22.3 | - | 5.3 | any site, Lumbal spine, total hip | low BMD |
| Shaharir  et al. 2016 | 424 | 38.9 | 91.9 | - | 0.8 | - | - | - | - | 29.7 | 9.2 | any site | osteoporosis |
| SINIGAGLIA et al. 1999 | 84 | 30.5 | 100 | 5.7 | 1.13 | 21.5 | 0 | - | 23.1 | 23.5 | 7 | any site | osteoporosis |
| So et al. 2011 | 21 | - | 100 | - | - | - | - | - | - | - | - | any site | low BMD, osteoporosis, osteopenia |
| Souto  et al. 2012 | 159 | 42.1 | 94.3 | - | - | - | 44.0 | - | - | - | 13.0 | any site, Lumbal spine, femoral neck，total hip | low BMD, osteoporosis, osteopenia |
| Sun  et al. 2015 | 119 | 32.6 | 100 | 11.0 | - | 20.6 | 11.9 | - | - | - | 1.8 | any site | low BMD, osteoporosis, osteopenia |
| Tang  et al. 2013 | 180 | 42.3 | 100 | 2.6 | 0 | 22.5 | 39.0 | - | 18.6 | - | 10.7 | total hip, lumbar spine | osteoporosis |
| TANG  et al. 2012 | 78 | 45.3 | 100 | 2.0 | 1.0 | 22.5 | 53.8 | 100 | 22.3 | - | 13.6 | any site | low BMD, osteoporosis, osteopenia |
| Toloza  et al. 2010 | 117 | - | 100 | 4.9 | 2.0 | - | 52.4 | - | - | 30.6 | - | any site | low BMD, osteoporosis, osteopenia |
| UARATANAWONG et al. 2003 | 74 | 31.8 | 100 | 2.9 | 2.1 | 22.7 | - | 100 | 5.2 | - | 2.5 | lumbar spine | low BMD, osteoporosis, osteopenia |
| Yeap  et al. 2009 | 98 | 30.1 | 100 | 8.5* | - | - | 0 | - | 9.1* | 24.9 | 3.0* | any site | low BMD, osteoporosis, osteopenia |
| Yee  et al. 2004 | 242 | 39.9* | 95.5 | - | - | - | 45.5 | - | - | - | 7.0* | any site | low BMD, osteoporosis, osteopenia |
| Zhu  et al. 2014 | 127 | 46.9 | 100 | 1.0 | 1.0 | - | 82.0 | - | - | - | 10.4 | femoral neck, lumbar spine,total hip | low BMD, osteoporosis, osteopenia |
| Zurek et al. 2003 | 18 | - | 100 | - | - | - | 32.7 | - | - | - | - | lumbar spine, neck of femur | low BMD, osteoporosis, osteopenia |
| Zurek et al. 2010 | 51 | 42.2 | 100 | - | - | - | 43.1 | - | - | - | - | lumbar spine, proximal femur, distal forearm | low BMD, osteoporosis, osteopenia |

*Median. SLEDAI=Systemic Lupus Erythematosus Disease Activity Index; BMD=bone mineral density; SLICC/SDI=Systemic Lupus International Collaborating Clinics/ACR Damage Index; BMI=Body mass index; GC=Glucocorticoid.
